# A novel male sterility-fertility restoration system in plants for hybrid seed production

**DOI:** 10.1038/srep11274

**Published:** 2015-06-15

**Authors:** Surendra Pratap Singh, Sudhir P. Singh, Tripti Pandey, Ram Rakshpal Singh, Samir V. Sawant

**Affiliations:** 1 Plant Molecular Biology Laboratory, CSIR-National Botanical Research Institute, Rana Pratap Marg, Lucknow, 226001, INDIA; 2 Academy of Scientific and Innovative Research (AcSIR), Anusandhan Bhawan, 2 Rafi Marg, New Delhi-110 001, India; 3 National Agri-Food Biotechnology Institute, Department of Biotechnology (DBT), C-127, Industrial Area, Phase VIII, Mohali, 160071, INDIA; 4 Department of Botany, University of Lucknow, Lucknow-226007, INDIA

## Abstract

Hybrid seeds are used for stimulated crop production, as they harness heterosis. The achievement of complete male-sterility in the female-parent and the restored-fertility in F1-hybrids are the major bottlenecks in the commercial hybrid seed production. Here, we report a male sterility–fertility restoration system by engineering the inmost nutritive anther wall layer tapetum of female and male parents. In the female parent, high–level, and stringent expression of Arabidopsis autophagy–related gene *BECLIN1* was achieved in the tapetum, which altered the tapetal degeneration program, leading to male sterility. This works on our previously demonstrated expression cassette based on functional complementation of TATA-box mutant (TGTA) promoter and TATA-binding protein mutant3 (TBPm3), with modification by conjugating Long Hypocotyle in Far-Red1 fragment (HFR1^NT131^) with TBPm3 (HFR1^NT131^-TBPm3) to exercise regulatory control over it. In the male parent, tapetum–specific Constitutive photo-morphogenesis1 (COP1) was expressed. The F1 obtained by crossing these engineered parents showed decreased *BECLIN1* expression, which was further completely abolished when COP1-mutant (COP1^L105A^) was used as a male parent, leading to normal tapetal development and restored fertility. The system works on COP1-HFR1 interaction and COP1–mediated degradation of TBPm3 pool (HFR1^NT131^-TBPm3). The system can be deployed for hybrid seed production in agricultural crops.

Hybrid crops have been contributing to the substantial global rise in agricultural output over the past few decades as they harness heterosis (hybrid vigor), a phenomenon of outperformance of F1 hybrid progeny compared with their parents in terms of yield, biotic and abiotic resistance^1^. Their utilization offers a 20% to more than 50% yield increase^2^ and contributes to more than half of the production of the major crops^3^. Precise control over pollen fertility in the female parent and the fertility restoration in F1 hybrids are the prerequisites in the commercial production of the F1 hybrid in self-pollinating crops^4^. Restoration of male fertility in hybrids is especially important in crops where the desired agricultural products are seeds, such as cereals, pulses and so on.

Several approaches have been explored to limit self-fertilization in the female parent line for the development of an effective hybrid seed production system, such as emasculation (manual removal of male reproductive organs), chemical-induced male sterility, cytoplasmic and nuclear male sterility, and biotechnological approaches for pollen abortion. Emasculation involves labor-intensive and time-consuming processes for large-scale hybrid seed production. The use of chemicals is limited by the issues related to bio-safety, variable effects, optimum dose and cost effectiveness^5^. The cytoplasmic and nuclear male sterility is limited by maintenance of multiple lines, partial and unstable male sterility and limited fertility restoring gene sources, all of which restrict the economic benefits of the hybrid^6^. A number of biotechnological strategies have been deployed to restrict self–fertilization in plants^7^. Several of the transgenic systems possess the male fertility–restoring constituent^8^,^9^,^10^,^11^,^12^,^13^,^14^,^15^,^16^,^17^. However, the only commercialized transgenic male sterility method is SeedLink^TM^, which relies on the expression of bacterial cytotoxic ribonuclease (Barnase) in the male reproductive organ of the female parent line and fertility restoration by ribonuclease inhibitor (Barstar) delivered by the male parent^18^,^19^. Barnase-barstar system is tested in many crops^20^; however, issues such as leaky expression of the barnase gene and difficulty in obtaining restoration lines of the barstar gene^9^,^20^,^21^,^22^,^23^ and biosafety concerns associated with the use of the bacterial cytotoxic gene in food crops are the key challenges associated with its applicability. Hence, it is desirable to develop a hybrid seed system that is equipped with capabilities of complete pollen abortion in a biologically safe and tightly controlled manner, as well as efficient male fertility restoration in the F1 hybrid.

Tapetum is the sporophytic tissue and innermost wall layer of the micro-sporangia in the angiosperm plants^24^. It plays an important role in the development of male gametophyte (microspore) by providing enzymes, nutrients and wall material, first by secretion and eventually by degeneration^25^. Tapetal degeneration is a programmed cell death (PCD) event^26^,^27^ with typical cytological features of cell shrinkage, mitochondria and cytoskeleton degeneration, nuclear condensation, oligonucleosomal cleavage of DNA, vacuole rupture, and endoplasmic reticular swelling^26^,^28^,^29^,^30^,^31^. Tapetal PCD at a specific developmental stage is crucial for pollen fertility, and disruption of the timing of PCD, either early or delayed, results in pollen abortion or male sterility. Several transgenic approaches have been developed to generate male sterile plants either through early tapetum degeneration by expressing *BARNASE*^18^, *RNase* -*1*^19^, *DIPTHERIA TOXIN-A*^32^, *RIBOSOME INACTIVATING PROTEIN*^33^, and *BAX*^28^, or by delaying tapetal PCD through *BAX INHIBITOR*^28^, ethylene receptor gene Cm-ETR1/H69A^34^, cystein protease BoCysP1 and BoCP3^35^.

Here, we established a transcription regulation system for male sterility–fertility restoration in plants. It includes two tapetum-specific expression cassettes; one expressed in the female parent and the other expressed in the male parent of the desired F1 progeny. The female expression cassette has the potential to attain high-level, stringent expression of a desired gene limited to tapetal cells, but the expression switches to abolition in F1 when regulated by the male component. We successfully deployed this system to express the desired gene (we earlier demonstrated for male sterility) Arabidopsis *BECLIN1*^15^, which generates the complete male sterile parent by altering the tapetal degeneration program. The male fertility of F1 progeny was completely restored due to the abolished expression of *BECLIN1*,> as the tapetal degeneration program was sustained to be normal. The female component works on the functional complementation of mutated TATA-box (TGTA) and TBPm3^36^ with aided HFR1^NT131^ fused to TBPm3 (HFR^1NT131-^TBPm3). The abolition of *BECLIN1* expression in F1 was achieved by limiting TBPm3 through COP1 (male component) –mediated degradation^37^,^38^,^39^ of the fusion protein HFR1^NT131-^TBPm3. *Nicotiana tabacum* has been used for the proof of principle presented here, but the essential elements of the technology are generic and possibly will work in other crops.

## Results

### High-level, stringent and spatio-temporal expression of the desired gene in postmeiotic tapetum

**Figure 1:**
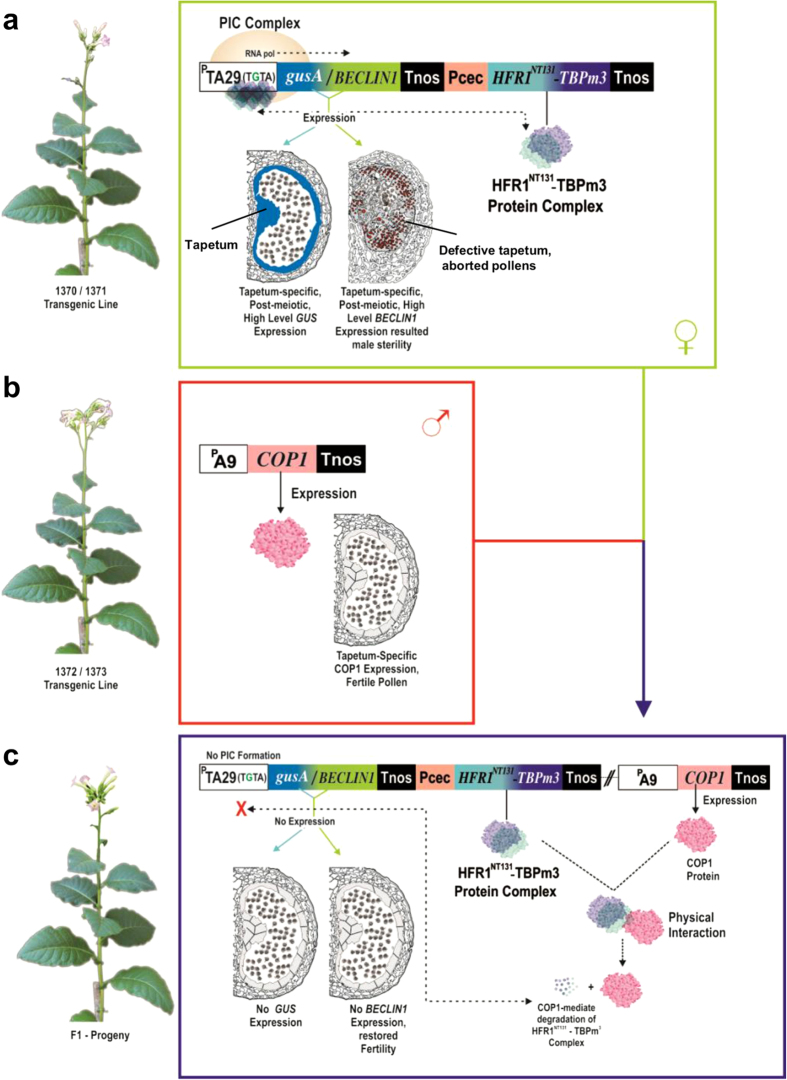
Experimental design and underlying mechanism of the male sterility–fertility restoration system in plants for hybrid seed production. (**a**) Generation of male-sterile female parent for hybrid breeding. The female expression cassette (1370/1371) contains two transcriptional units: expression component (^P^TA29_(TGTA)_-*gusA/BECLIN1*-Tnos) and the regulatory component (^P^Pcec-*HFR1*^*NT131*^*-TBPm3*-Tnos). The regulatory component express conjugated protein HFR1^NT131^-TBPm3, which binds to TATA-box mutated promoter (^P^TA29_(TGTA)_) of expression component, and resulted transcriptional pre-initiation complex (PIC) formation. The high-level and tapetum-specific expression of *gusA* or *BECLIN1* was achieved, as TGTA-mutated promoter was functionally complemented by TBPm3 (or HFR1^NT131^-TBPm3) pool. This expression cassette is useful in achieving tapetum-specific, high-level expression of the male-sterility gene (we expressed *BECLIN1*). (**b**) The male expression cassette (1372/1373) expresses COP1 (or COP1^L105A^) by using the tapetum-specific promoter A9, giving normal male-fertile plants. (**c**) Fertility restoration mechanism of F1. F1 is obtained by crossing with plants expressing female (male-sterile) and male (male-fertile) expression cassettes. Regulatory component express HFR1^NT131^-TBPm3 and male-component express COP1 (or COP1^L105A^) proteins in the F1-tapetal cell. The COP1 physically interacts with the HFR1^NT131^ fragment of the conjugated protein (HFR1^NT131^-TBPm3) and sequentially degrades it, resulting in the unavailability of TBPm3, hence no PIC formation on the TGTA-mutated TA29 promoter, leading to expression abolition of gusA or *BECLIN1*. *BECLIN1* abolition resulted normal tapetal degeneration program and restored fertility of F1-progeny.[TA_29(TGTA)_ = Tapetum-specific promoter from tobacco with mutated TATA box to TGTA, *gusA*= β-glucuronidase, Pcec= artificial promoter^48^, *HFRI*^*NT131*^ = N-terminus 131 amino acid fragment of Long **h**ypocotyle in **f**ar **r**ed **1** (HFR1), *TBPm3* = TATA-binding protein with three amino acid substitution (Ile_152_ to Phe_152_, Val_161_ to Thr_161,_ and Leu_163_ to Val_163_), Tnos= transcriptional terminator, A9= tapetum-specific promoter from *Arabidopsis*, COP1= **Co**nstitutive **p**hotomorphogenic**1,** COP1^L105A^= COP1-mutant with increased nuclear localization^38^]

**Figure 2:**
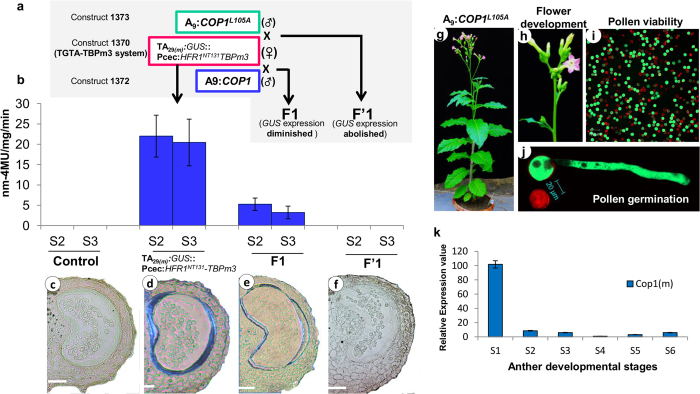
Analysis of reversible expression system. **(a)** The strategy of crossing; the development of F1^s^, transgenic plants of 1370 was taken as ♀-parent and was crossed with 1372 and 1373 as ♂-parent, resulting in F1 (1370_(♀)_ × 1372_(♂)_) and F’1 (1370_(♀) _× 1373_(♂)_) progeny, respectively. **(b)** GUS expression analysis in ♂-parent (1370), F1 and F’1 hybrids at anther developmental stages 2 and 3; the expression values are normalized with the control (NTPH). The error bar represents SD of n = 10 independent line. **(c–f)** Gus staining in the stage 2 anther of the control **(c)**, 1370 transgenic line **(d)**, and F1 **(e)** and F’1 **(f)** lines. Bar = 100 μm. **(g)** Transgenic plant of 1373 (^P^A9:*COP1*^*L105A*^) showing normal growth and development. **(h)** Inflorescence of 1373 transgenic lines showed normal flowering and flower development.**(i)** Pollen viability using FDA-PI method; green fluorescence of FDA shows viable pollens whereas red fluorescence of PI indicates aborted pollens. Bar = 100 μm. (FDA= fluorescein diacetate, PI= propedium iodide). **(j)** *In-vitro* pollen germination and staining with FDA and PI, germinated pollens with pollen tube whereas abortive pollen with PI staining and no germination. Bar = 20 μm. **(k)** Bar graph showing the relative expression of COP1^L105A^ in developing anthers of stages 1–6. Total RNA was isolated from anthers of the transgenic lines (1373) for qRT-PCR assay (n = 3 independent biological repeats). UBIQ10 was taken an internal control. Error bars indicate standard deviation (SD).

We have previously utilized TATA box binding protein mutant3 (TBPm3) and TATA-box mutation TGTA complementation system^36^ for high-level and stringently regulated expression in anther tapetum^15^. The expression through promoters with TATA-box mutated to TGTA was not driven by a native TBP protein because of its altered affinity^40^,^41^,^42^, however, three amino-acid substitution mutant (Ile_152_ to Phe_152_, Val_161_ to Thr_161,_ and Leu_163_ to Val_163_) of TBP (TBPm3) complement the TGTA-mutation and drive the expression from such promoters^43^,^44^. The high-level expression of the TGTA-TBPm3 complementation system was achieved due to a dedicated pool of TBPm3 protein to TGTA-containing promoters^15^,^36^,^45^. In this work, we seek transcriptional control over the TGTA-TBPm3 complemented system by fusing TBPm3 with the 131 amino-acid N-terminal domain of HFR1 (HFR1^NT131^) and specifically targeting it for degradation by COP1, thus limiting TBPm3 protein in the cell. The limited cellular availability of TBPm3 protein leads to the abolition of expression of the transgene in tapetum cells (Fig. 1a–c). Tapetum-specific promoter TA_29_ (^P^TA_29_)^46^ was utilized to construct a TGTA-TBPm3 complemented system to attain high-level and stringent expression, specific to the postmeiotic tapetum as previously described^15^, with a modification of fusing *HFR1*^*NT131*^ at the N-terminus of *TBPm3* to excercise further control over it (Supplementary Fig. S1-a-I). The transgenic lines of *Nicotiana tabacum* cv. Petit Havana (NTPH) harboring this expression cassette 1370 (carrying TGTA-driven *gusA* gene and *HFR1*^*NT131*^*:TBPm3* fusion in a single T-DNA cassette; Supplementary Fig. S1-a-I) were normal during growth and development with a similar range of pollen viability, germination, and seed setting when compared with NTPH plants (Supplementary Fig. S1-b and c). The anther development has been divided into seven premeiotic (−7 to −1; negative sign represents stages before the meiosis) and twelve postmeiotic stages (1 to 12) in tobacco^46^. The expression of gusA protein was examined in the anthers of stages 2 and 3 in 1370 transgenic lines. The gusA expression in the stage 2 anther ranges from 11.45 nmoles mg^−1^min^−1^ to 47.57 nmoles m^−1^min^−1^ (Supplementary Fig. S2-a), with an average expression of 22.00 ± 5.15 nmoles mg^−1^min^−1^ (Fig. 2b); at stage 3, the expression ranges from 10.45 nmoles mg^−1^min^−1^ to 45.65 nmoles mg^−1^min^−1^(Supplementary Fig. S2-b), with average expression being 20.43 ± 5.74 nmoles mg^−1^min^−1^(Fig. 2b) in the anthers of transgenic lines evaluated. Histochemical GUS staining confirmed that the expression of gusA protein was limited to tapetal cells in anthers (Fig. 2d). Expression was not detected in other plant organs such as leaves, emasculated flower buds, stems, and roots (data not shown). Taken together, our results showed that high-level and stringent expression was achieved in the postmeiotic (stages 2 and 3) tapetum, using the expression vector 1370.

### Transcriptional abolition of TGTA-TBPm3 system based on HFR1^NT131^-COP1^L105A^–mediated light signalling

To evaluate whether limiting cellular protein levels of TBPm3 can abolish *gusA* expression, we developed transgenic tobacco lines expressing *COP1* under the control of Arabidopsis tapetum-specific promoter A9 (^P^A9)^47^. In construct 1370, the desired gene (*gusA* in the case of 1370 cassette) was expressed under the control of the TGTA-mutated tapetum-specific promoter, and the translationally fused HFR1^NT131^-TBPm3 complex was under the regulation of the Pcec promoter^48^. *HFR1*^*NT131*^ is a truncated form (NT131: N-terminus 131-amino-acid fragment) of Long Hypocotyle in Far Red1 (HFR1), a basic helix-loop-helix (bHLH) involved in the seedling response to far-red light. The NT131 amino-acid fragment loses its bioactivity but retains its interaction ability with another protein COP1 (Fig.1a–c). COP1 is a repressor of light-mediated photo-morphogenesis that physically interacts with HFR1 (and HFR1^NT131^) and mediates its targeted degradation through the proteosome^37^,^39^,^49^,^50^. We, therefore, postulated that the fusion protein HFR1^NT131^:TBPm3 will also be degraded by COP1 and this was exploited to exercise control over the TGTA-TBPm3 complementation system (construct 1370). To achieve this, a regulatory expression cassette (1372) was designed, in which the Arabidopsis *COP1* gene was placed under the control of the Arabidopsis tapetum-specific promoter A9 (^P^A9)^47^ (Supplementary Fig. S1-a-III). The tobacco transgenic lines of 1372 were normal during growth and development with normal flowering, pollen viability, *in*-*vitro* pollen germination and seed setting similar to those of the control (NTPH) plants (Supplementary Fig. S1b-c, and S3a-d). The relative expression of *COP1* was evaluated using qRT-PCR in anther developmental stages 1 to 6. The highest expression was found in the stage 1 anthers (Supplementary Fig. S3-e). To screen the best expressing transgenic lines, relative expression of *COP1* was compared using qRT-PCR in the stage 1 anthers in 19 transgenic lines. The transgenic lines 13 and 33 were selected for further studies based on their higher expression (Supplementary Fig. S4-a). To validate our hypothesis of achieving transcriptional control over the TGTA-TBPm3 system by COP1-mediated degradation of HFR1^NT131^-TBPm3, crossing was performed between female 1370 lines (L3 and L5; Supplementary Fig. S2-a) and male 1372 plants (T13 and T33; Supplementary Fig. S4-a), raising F1 progeny. The F1 seeds were harvested, subjected to double antibiotic selection of kanamycine and hygromycine, considering the selectable marker genes of the constructs 1370 (*NPTII*) and 1372 (*HPTII*), and further confirmed by PCR (Supplementary Fig. S5a–c). The fluorimetric GUS analysis revealed a 4–6–fold reduced GUS activity in the anthers of F1 plants during stages s2 and s3 as compared to 1370 plants (Fig. 2b, Supplementary Fig. S2-c and d). Histochemical GUS staining of F1 plants revealed that the GUS expression was stringent to tapetal cells but diminished when compared with 1370 (Fig. 2e) Thus, COP1 imparts its control over the TGTA-TBPm3 system that is mediated by HFR1^NT131-^ linked TBPm3 protein (HFR1^NT131^-TBPm3) degradation in F1 plants, albeit the expression was not completely abolished. The one reason for incomplete control of COP1 could be its light-responsive nuclear and cytoplasmic localiszation.

To develop a fertility restoration system, it is necessary to completely abolish the expression of the male sterility gene (here, *BECLIN1*) in the F1 hybrid. We postulate that increased nuclear localization of COP1 should cause complete degradation of HFR1^NT131^-TBPm3, resulting in its unavailability to form the transcriptional pre-initiation complex (PIC) on the TGTA-mutated TATA-box of the ^P^TA_29_ promoter, leading to expression abolition in F1 progeny. To achieve it, COP1 was mutated to COP1^L105A^. The mutation in COP1 (COP1^L105A^) was reported to increase nuclear localization but it retained its normal functioning^38^. The expression cassette 1373 (Supplementary Fig. S1-a-iv) was designed to express mutated *COP1* (*COP1*^*L105A*^) using ^P^A9. The transgenic plants expressing *COP1*^*L105A*^were found to be normal during growth, development, and fertility (Fig. 2g–j, Supplementary Fig. S1a-b). The highest *COP1*^*L105A*^ expression was found at the s1 anther development stage (Fig. 2k). Fifteen transgenic lines were compared for relative expression; lines 3 and 5 were selected for the crossing experiment based on their higher expression (Supplementary Fig. S4-b). The cross was made between female 1370 (emasculated) and male 1373 to raise F’_1_ seeds. The F’_1_ seeds were screened on double antibiotic selection medium (Kanamycin and Hygromycin) and standard PCR. The fluorimetric and histochemical GUS analysis of anthers of F’_1_ plants at developmental stages s2 and s3 showed completely abolished expression of gusA protein (Fig. 2b,f, and Supplementary Fig. S2e,f). The results indicated that the nuclear localization of COP1^L105A^ imparts its complete control over the TGTA-TBPm3 system through HFR1^NT131^-mediated degradation of TBPm3.

### Complete male sterility by high-level expression of *BECLIN1* in tapetum cells

**Figure 3:**
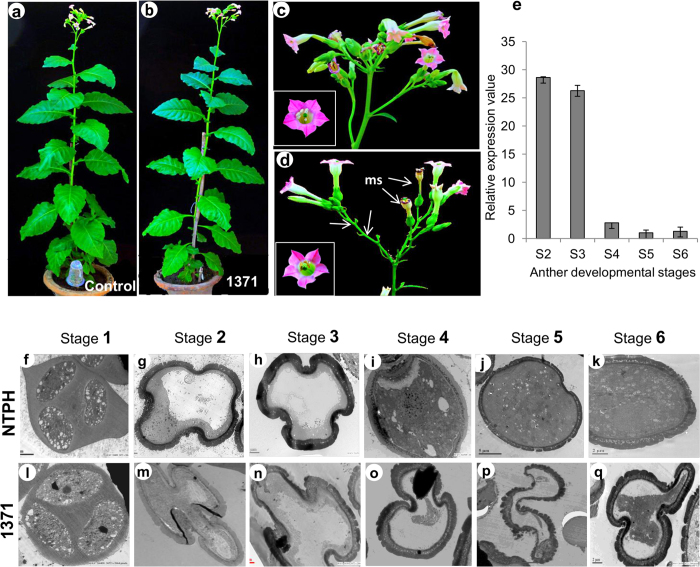
Analysis of BECLIN1 expressing transgenic lines. **(a)** Control (wild type) and **(b)** 1371 transgenic plants. Inflorescence, flower and seed setting in control (**c**) and 1371 transgenic plants (**d**). The control and 1371 plants showed normal growth and development except male sterility in 1371 transgenics (arrows indicate the dying and detachment of flower that are unsuccessful in fertilization due to male sterility). **(e)** qRT-PCR for *BECLIN1* expression in 1371 transgenic lines of anther at stages 2 to 6, highest expression was found in stage 2 and 3 of anther development. Error bars represent SD of three (n = 3) biological replicates. Transmission electron micrograph (TEM) of pollen of anther stage 1 to 6 in control **(f–k)** and in 1371 transgenic plants **(l–q).** Bar = 5 μm.

We previously demonstrated that high-level expression of Arabidopsis *ATG6/BECLIN1* in the tapetum under control of the TGTA-TBPm3 complementation system leads to complete male sterility in tobacco^15^. To develop a male sterility–fertility restoration system, we cloned Arabidopsis *ATG6/BECLIN1* into the 1370 vector replacing *gusA*, thus developing the construct 1371 (Supplementary Fig. S1-a-ii). The tobacco transgenic lines expressing 1371 showed no morphological anomaly, with normal growth and development comparable to wild plants except male sterility (Fig. 3a,b). The flowers of 1371 transgenics showed healthy stigma and other accessory whorls, whereas pollen grains laden to the anther were very low (Fig. 3c,d). The flowers failed to self-fertilize because of the formation of non-viable pollen, which eventually dries out and drops from the floral axis (Fig. 3d arrow shows point of detachment and drying); whereas when fertilized with the viable pollen of wild plants, a normal seed setting was observed (data not shown). The results suggest that stigma receptivity and female reproductive organs of 1371 transgenics were normal, and the drop of flowers was due to the development of aborted pollen and failure in fertilization.

The relative expression of Arabidopsis *BECLIN1* was compared using qRT-PCR at a different anther developmental stage of the 1371 transgenic lines; the highest expression was found in stages 2 and 3 (Fig. 3e). To understand the effect of postmeiotic, tapetum-specific, and high-level expression of *BECLIN1* on the pollen development, transmission electron microscopy (TEM) of developing pollens was performed. No obvious differences were found till stage 1, between 1371 transgenics and control (NTPH) as meiosis was normal and microspore tetrad was surrounded by the callose in both control (Fig. 3f, Supplementary Fig. S6-b) and in transgenic (Fig. 3l, Supplementary Fig. S6-i) plants. At stage 2, microspores became free due to callose decomposition, the nucleus was displaced aside due to a single large vacuole and development of the orderly microspore exine structure was seen in the control (Fig. 3g, Supplementary Fig. S6-c); whereas in 1371 transgenics the tetrad was not fully separated due to remnants of the callose deposition and deformation had started in microspores (Fig. 3m, Supplementary Fig. S6-j). The vacuole gets enlarged with demarked wall formation in stage 3 microspores in case of control (Fig. 3h, Supplementary Fig. S6-d), whereas deformation increases in 1371 transgenics (Fig. 3n, Supplementary Fig. S6-k). At stage 4, the generative cell was attached to the wall with a prominent vegetative nucleus, cytoplasm, and normal exine and intine development in the control (Fig. 3i, Supplementary Fig.S6-e); whereas in 1371 transgenics, the cytoplasm was collapsed and pollen wall deformations were visible (Fig. 3o, Supplementary Fig. S6-e). The microspore shape became nearly circular and was filled with dense cytoplasm and small vesicles; the pollen wall also differentiated into well-developed exine and intine at stages 5,6 in control pollens (Fig. 3j,k, Supplementary Fig. S6f-g); whereas in case of pollens of 1371 transgenics, the cytoplasm collapsed and severe deformity was observed in the pollen wall (Fig. 3p,q, Supplementary Fig. S6m-n).

**Figure 4:**
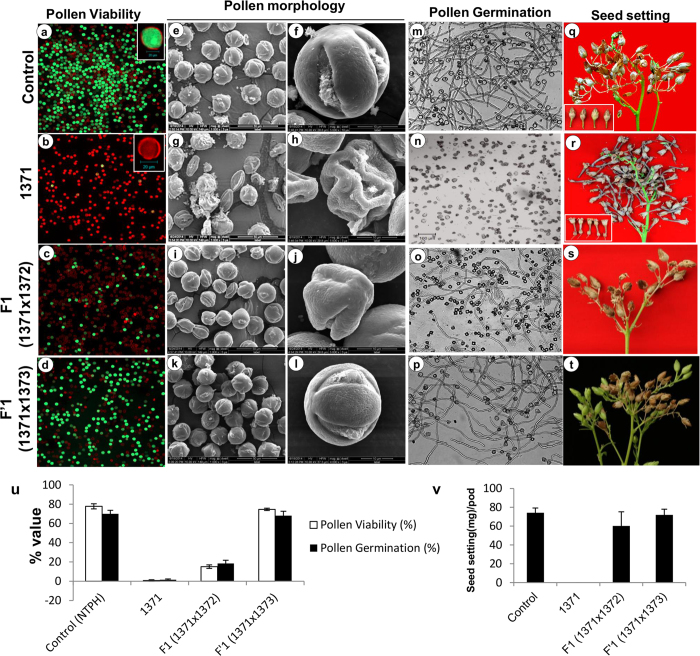
Transgenic male sterility and fertility restoration in tobacco. **[a–d]** Pollen viability assay using FDA-PI method in control (**a**) 1371 transgenic (**b**) F1 obtained by crossing (1371_(♀)_ × 1372_(♂)_) (**c**) and F’1 (1371_(♀)_ × 1373_(♂)_) (**d**) lines. Bar = 100 μm, inset bar = 20 μm. **[e–l] Scanning electron microscopic (**SEM) analysis in control (e and f), 1371 transgenic (**g** and **h**), F1 (**i** and **j**), and F’1 lines (**k** and **l**). Bar = 50 μm in e, g, i, and k and 10 μm in **f,h,j**, and **l**. **[m–p]** *In-vitro* pollen germination assay in control (**m**), 1371 transgenic (**n**), F1 (**o**) and F’1 (**p**) lines pollens. Bar = 100 μm. **[q–t]** Seed setting in control (**q**) 1371 transgenic (**r**) F1 hybrid (**s**) and F’1 hybrid (**t**) lines. **[u]** Quantitative analysis (in %) of pollen viability and *in-vitro* pollen germination in control, 1371 transgenic, F1 hybrid and F’1 hybrid lines. Error bar represents SD of n = 10 lines. [**v**] Quantitative analysis (in mg/pod) of seed setting in control, 1371 transgenic, F1 hybrid, and F’1 hybrid lines. Error bar represents SD of n = 10 lines.

We next examined the pollen viability, *in-vitro* pollen germination and seed setting in wild and 1371 transgenics. The 1371 transgenic lines showed considerably low pollen viability (Fig. 4a,b,u) with nil germination (Fig. 4m,n,u) as compared to the control (wild type). In control plants, bagging of a young inflorescence before anthesis results in normal seed setting (Fig. 4q,v), unlike in male sterile plants in which all the bulbs were seedless (Fig. 4r,v). Further, the scanning electron micrograph (SEM) of stage 7 pollen of control showed normal pollen morphology (Fig. 4e,f, and Supplementary Fig. S7a-b); whereas deformed pollens were seen in the 1371 transgenic lines (Fig. 4g,h, and Supplementary Fig. S7c-d). The results obtained by the new system are in agreement with our previous finding^15^ that overexpression of *BECLIN1* in the anther tapetum resulted in complete male sterility in the tobacco transgenic plants. In addition, the male sterile female lines are equipped with additional regulatory control over the TGTA-TBPm3 system operated through the male component.

### Complete restoration of male fertility in F1 hybrid

**Figure 5:**
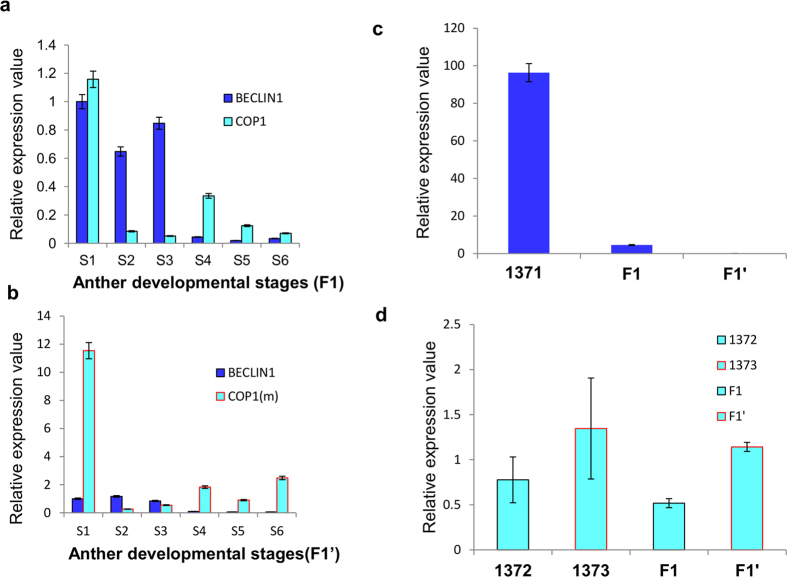
Expression analysis. [**a**] Bar diagram showing the expression of *BECLIN1* and *COP1* in the anther stages 1 to 6 in F1 lines (1371_(♀)_ × 1372_(♂)_). Total RNA was isolated from the anther of stages 1 to 6 for qRT-PCR assay (n = 3 independent biological repeats). UBIQ10 was used as an internal control. *BECLIN1* expression value at stage 1 was set as 1, and the relative gene expression levels were calculated. Error bars indicate SD. **[b]** Bar diagram showing the expression of *BECLIN1* and *COP1*^*L105A*^ in the anther stages 1–6 in the F’1 lines (1371_(♀)_ × 1373_(♂)_). Total RNA was isolated from the anther of stages 1–6 for qRT-PCR assay (n = 3 independent biological repeats). UBIQ10 was used as an internal control. *BECLIN1* expression value at stage 1 was set as 1, and the relative gene expression levels were calculated. Error bars indicate SD. **[c]** Bar diagram showing the expression of *BECLIN1* in the anther stage 3 of 1371, F1 and F’1. **[d]** Bar diagram showing the expression of *COP1* and *COP1*^*L105A*^ in the anther stage 1 of 1372, 1373, F1 and F’1 plants.

Restoration of pollen fertility in F1 progeny is a prerequisite for hybrid seed production. The male sterile 1371 transgenic female lines were crossed with the selected male lines expressing either COP1 (1372) or COP1^L105A^ (1373) to raise F_1_ and F’_1_ progeny, respectively (Supplementary Fig. S5). In F1 [1371(♀) × 1372(♂)] plants, pollen viability was recorded ~15.14% (Fig. 4c,u) as compared with ~77.71% of control; whereas *in-vitro* pollen germination was ~18.5% (Fig. 4o,u) in F1 as compared with ~70% in case of control plants. The pollen viability of F1 was low, but the seed setting was found to be comparable to the control plants (Fig. 4s,v). The SEM of stage 7 pollens showed a high population of aborted and deformed pollens with a few normal architecture pollens in F1 plants (Fig. 4i,j, Supplementary Fig. S7 e-f).The relative expression of *BECLIN1* and *COP1* was quantified in s1-s6 anther developmental stages of F1 plants. We found higher accumulation of *AtBECLIN1* transcript in s1, s2, and s3 stages. The *COP1* expressed at considerably high level in the s1 stage. We next compared the expression of *BECLIN1* in s2 stage in F1 with that of 1371 transgenic male sterile lines and found about ~21-fold decrease in the expression (Fig. 5a,c,d). The results, thus, indicate that COP1 (1372) mediates partial restoration of fertility in F1 plants by reducing the expression of *BECLIN1*. In F’1 [1371(♀) × 1373(♂)] progeny, the pollen viability and pollen germination was found to be comparable to that of control plants (Fig. 4d,p,u). The SEM of stage 7 pollen showed pollen architecture similar to control pollen (Fig. 4k,l, and Supplementary Fig. S7g-h). The seed setting was normal with seed weight ~78.89 mg/pod, comparable to control ~74.23 mg/pod (Fig. 4t,v). The expression of *BECLIN1* was completely abolished, while the expression of *COP1* was found to be the highest in the s1 anther stage in F’1 (Fig. 5b–d). The results established complete restoration of male fertility in F’1 plants by utilizing the principle of COP1-mediated degradation of HFR1^NT131^-TBPm3 and by abolishing the expression of *BECLIN1*.

### BECLIN1 expression resulted in abnormal tapetal growth and delayed degeneration

**Figure 6:**
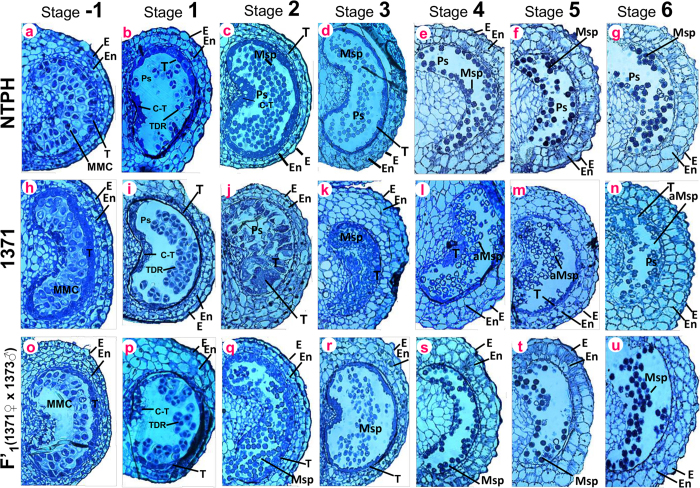
Transverse anatomical comparison of anther development in wild type (NTPH), Beclin1 transgenic (1371) and F’1 Semi-thin cross sections of control ([**a**] to [**g**]), 1371 transgenic ([**h**] to [**n**]), and F’1 ([**o**] to [**u**]) at anther stages from −1 to stage 6. **E**, epidermis; En, endothecium; ML, middle layer; T, tapetum; TDR, tetrads; Msp, microspore; Ps, pollen sac; **C-T**, connective tapetum, MMC, microspore mother cell; aMsp, aborted microspore. Bars = 100 μm.

To determine the cause of pollen abortion in *BECLIN1* expressing transgenics (1371), we examined cross-sections of anther from control (NTPH) and 1371 transgenic lines at different anther developmental stages^46^. At stage −1, the tapetum was well developed enclosing microspore mother cells (MMC) which were in the stage of meiotic division in the control (Fig. 6a). At stage 1, the meiotic division occurs, forming dyad and tetrad with distinct tapetum in the control (Fig. 6b). However, no obvious differences were observed in the 1371 transgenic anther in stages −1 and 1, when compared with the control (Fig. 6h,i). The morphological defects started surfacing at stage 2; at this stage, well-developed tapetal layer enclosing nascent-free microspores were observed in control anther (Fig. 6c), whereas in 1371 anther, tapetal cells showed abnormal growth and the entire pollen sac was filled with debris and unstructured cell matter (Fig. 6j). At stage 3, control tapetal cells shrank and microspores were clearly visible (Fig. 6d); whereas in 1371, the abnormal tapetal structure, debris, and unstructured cell matter filled the pollen sac and microspores were not clearly visible (Fig. 6k). At stage 4, the control tapetum was degenerating and only epidermis and endothecial layers were visible as surrounding filled mature pollens (Fig. 6e). Interestingly in 1371 anther, tapetal layer remained intact with empty pollens in the pollen sac (Fig. 6l). At stages 5,6, the control tapetum was completely degenerated and pollens were mature with a darkly stained dense cytoplasm (Fig. 6f,g); whereas in 1371 transgenics, the tapetum was still visible as enclosing empty aborted pollens (Fig. 6m,n). To ensure that the abnormal tapetal growth and delayed degeneration in the male sterile parent was because of the expression of *BECLIN1*, the anther cross-sections of the F’1 were observed, as we had already observed complete abolition of *BECLIN1* expression in F’1. The cross-section of F’1 showed normal development of tapetum and pollen similar to the control (Fig. 6o–u), confirming that the tapetal abnormality and pollen abortion in 1371 were due to the expression of *BECLIN1*.

## Discussion

In this study, we have developed a male sterility–fertility restoration system for heterosis breeding in plants. In the female parent, tapetum-specific, high-level and postmeiotic expression of the Arabidopsis *BECLIN1/ATG6* gene led to complete male sterility. The tapetum–specific expression was completely abolished and male fertility was restored in the F1 hybrid.

The male sterility system (construct 1371; Supplementary Fig. S1a-I) is the modification of our previously reported two component system^15^ based on the principle of expression restoration of a TGTA-mutated promoter by providing a complementing TBPm3, which binds specifically to the TGTA^36^. The system gave several-fold enhancement of expression over the native tapetum-specific promoter (Fig. 1a), in agreement with our previous report^15^. In this study, we included the light-regulated transcription factor HFR1 that exhibits its COP1-mediated degradation^37^,^49^,^50^ to limit TBPm3 and abolish expression of the desired gene. HFR1 is a transcription factor (bHLH) consisting of 292 amino acids, of which the N-terminus 131 amino acid interacts with COP1 and the C-terminus 161 amino acid has a functional role to bind DNA and promote photomorphogenesis^39^,^49^. We fused the HFR1^NT131^ fragment to the N-terminus of TBPm3, thus making the fusion protein HFR1^NT131^-TBPm3. This fusion did not affect the functionality of TBPm3, resulting in a high-level expression of the desired gene (*gusA or BECLIN1*) (Fig. 1a). The transgenic plants expressing *gusA* were normal in growth and development, and the strength and stringency of the expression system were not compromised (Fig. 2b,c–f, and Supplementary Fig. S1-b and c). To ensure the transcriptional abolition of tapetum-specific expression in F1 progeny, it was crossed with the male parent expressing COP1 under the regulation of the Arabidopsis ^P^A_9_ promoter^47^ (Fig. 1b). The expression of COP1 ensures its availability to bind with HFR1^NT131^ and to degrade it in the F1 tapetal cell. The degradation of TBPm3 was also achieved, as it was conjugated HFR1^NT131^-TBPm3 (Fig. 1c). However, COP1 facilitates only partial abolition of the TGTA-TBPm3 complementation system in F1 (Fig. 2b,c–f). The COP1 protein is known to shuttle between the cytoplasm and nucleus^39^, and stoichiometrically insufficient nuclear concentration of COP1 might be a reason for partial expression reversion. Therefore, we made use of an alternative COP1-mutant (COP1^L105A^)^38^. The mutation retained dimerization and functional activity of the COP1 but increased its nuclear abundance. The expression of the reporter gene (*gusA*) was completely abolished in F’1 progeny when COP1^L105A^ lines were used as a male parent, instead of native COP1 (Fig. 2b,c–f). Thus, a tapetum-specific reversible expression system was established.

The male sterility and fertility restoration system developed by us are generic in nature and hence can be used with other reported genes for male sterility. Thus, the female expression cassette (1371) can be used to generate the male-sterile parent by expressing any known gene reported for the male sterility such as *BARNASE*^18^ , *BAX*^28^, and so on. The major advantage with our system that there is no specific requirement of the fertility restoration gene for example *BARSTAR* in *BARNASE*^18^,^19^. The male expression cassette (Construct 1373) offers restoration through abolishing transcription of the male sterility gene. We used the Arabidopsis *BECLIN1/ATG6*^15^ gene to raise complete male sterile transgenic plants. Genetic engineering of male-sterility and the fertility-restoration system have emerged as tangible options for hybrid seed production. Several restoration systems have been reported to redeem male fertility by inactivating the male sterility protein^18^,^19^, degrading transcripts of the male sterility gene^14^,^17^,^51^ and site-specific recombination in the male sterility gene^8^,^10^. However, efficient restoration of fertility has been discussed as one of the limiting factors in some of the systems^14^,^21^,^51^. The present system offers a system equipped with complete male sterility and fertility restoration in F1-progeny (Figs 4,1a–c; proposed model) with a novel approach.

The restoration of fertility of F1 hybrid is prerequisite, especially when the economic product is seed. The barnase/barstar system^18^,^19^ was deployed for the commercial hybrid production but the identification of efficient restorer (barstar) line was proven to be difficult in *Brassica juncea*; one in 54 cross-recombination between barnase (male sterile) × barstar (restorer) was adequately restored male fertility in the F1 hybrid^21^,^23^. Tapetum-specific promoter TA29 driven barstar (restorer) restore 65.6% male fertility (in terms of pollen viability), it was further improved to 78–90% when ^P^A9 and chimeric system were used to express barstar (restorer)^9^,^21^. Barnase weakly expressed in vegetative tissue resulted yield penalty in the plants^23^. The other barnase based systems; the Cre/loxp-mediated site-specific recombination system^10^, two-component system^22^, and split-gene system^4^,^11^ claimed 100% restoration of F1 fertility, however, use of toxic gene of trans-origin limited the acceptability due to biosefty concern in some countries. Pathogenesis-related (PR) β-1,3-glucanase gene based male sterility was only partially restored by pA9-driven sense and antisense PR glucanase fragments^51^. The temperature-sensitive *DIPTHERIA TOXIN-A* (*DTA*^*ts*^) confer conditional-male-sterility (18 ^°^C male sterility, 26 ^°^C restored fertility)^32^, and reversible male sterility in egg plant^16^ claimed complete restoration but works on ethanol inducible method which limit its practical applicability. In our system, female expression cassette (1371) expressing plants generate complete male sterility; when compared with control (pollen viability (%): 77.7 ± 1.3 (100%), pollen germination (%): 70 ± 3.6 (100%), and seed-setting (mg/pod): 74.23 ± 5 (100%)), 10 randomly selected *BECLIN1* expressing transgenic lines showed pollen viability (%): 0.76 ± 0.78 (~0.96%), pollen germination (%): 0.76 ± 0.73 (~2.3%) and nil seed setting (Fig. 4u,v). The fertility restoration of F1 progeny works on transcription abolition of male sterility gene (*BECLIN1*) regulated through male parent. When COP1 expressing lines (1372) were used as male parent, F1 showed ~21-fold reduction in *BECLIN1* expression (Fig. 5c), which restored pollen viability (%): 15.14 ± 1.8 (20%), pollen germination (%): 18.5 ± 3.3 (26%) that is sufficient for optimal seed setting (mg/pod) 60.2 ± 15 (81%) (Fig. 4u,v). We observed further improvement in fertility restoration when COP1-mutant (COP1^L105A^) lines (1373) were taken as male parent, which completely abolished the expression of *BECLIN1* resulting complete restoration of pollen fertility in F’1 with pollen viability (%): 74.58 ± 1.2 (96%), pollen germination (%): 69 ± 4.6 (~97.14% ) and seed setting (mg/pod) 71.9 ± 6.1 (97%) (Fig. 4u,v) comparable to the untransformed control plants.

Maintaining the male-sterile female lines is a prerequisite for future commercial application of this technique, and the genetic design of the male-sterile female line (construct 1371, Fig. S1a-ii) provides this opportunity. Crossing the heterozygous male-sterile female parent (BECLIN1/−) with its wild type (−/−) results in ~50% of the male-sterile progeny (1:1 ratio, (BECLIN1/−) and (−/−)). In future approaches, linking the herbicide resistance gene in the construct 1371 (as in SeedLink^TM^) will enable the selection of male-sterile female parents. However, this requires overplanting and eliminating half of the sown plants by applying herbicide to obtain pure male-sterile female parents.

In conclusion, we have developed a system for tapetum-specific, high-level expression of the desired gene to achieve complete male-sterility, along with a system for transcriptional control over the expression system for fertility-restoration in the F1-hybrid. The tapetum-specific expression of the *BECLIN1/ATG6* gene facilitated complete male sterility, and COP1-mediated HFR1 degradation system was used for repression of transcription of *BECLIN1* followed by fertility restoration in the F1 hybrid. The proposed male sterility-fertility restoration system described here will be a valuable future contribution for exploiting hybrid vigor and commercial production of hybrid seed.

## Methods

### DNA Constructs, Transformation and transgenic development

The four expression cassettes were constructed in the plant expression vector pBI_101_ and modified pCAMBIA1300 (Schematically presented in Supplementary Fig. S1A). All the vector constructs that were transformed in *Agrobacterium* and transgenic tobacco plants were developed as previously described^15^.

### Site-directed mutagenesis

TGTATATG mutation was introduced into the TATATATG box region of the TA29 promoter using Quik Change XL Kit (Stratagene, http://www.stratagene.com) as previously described^15^. In COP1, the L105A mutation^38^ was introduced using site directed mutagenesis PCR was performed according to the manufacturer’s instructions. 5′TTCGCGGCCGATAAGGCAGCGAAG 3′ mutation was introduced into the 5′ TTCTTGCTCGATAAGCTATTGAAG 3′ region of the COP1 gene by using two sets of primers COPM_f1 5′gct tta ccc taa ttt cgc ggc ccg ata agc tat tga aga aaa ctt c 3′, COPM_r1 5′gtt ttc ttc aat agc tta tcg gcc gcg aaa tta ggg taa agc tg 3′ and COPM_f2 5′ taa ttt ctt gct cga taa ggc agc gaa gaa aac ttc agc tcg gc 3′, COPM_r2 5′ cga gct gaa gtt ttc ttc gct gcc tta tcg agc aag aaa tta gg 3′ primers, Clones were screened by DNA sequencing for the desired mutation using T3 and T7 primers.

### Fluorimetric and histochemical GUS assay

Fluorimetric GUS assay was performed as previously described by^36^. All results are an average of 10 independent lines with three independent experiments of T1 lines. Histochemical GUS analysis was performed as described by^15^.

### Pollen viability and germination assay

Pollen from the blooming stage of flowers was collected and incubated in Fluorescein Diacetate (FDA)—Propidium Iodide (PI) solution for [2 mg/ml (FDA) in acetone and diluted by 10% sucrose drop by drop until turning milky with 1 mg/ml PI (PBS) added to a final concentration] for 5 min. The pollens were centrifuged at 5000 rpm for 1 min, the supernatant was discarded, 3 washings were done in phosphate-buffered saline (PBS), and finally suspended in 50ul PBS. It was mounted on slowfade@antifade (Molecular probes) and observed under a confocal microscope (LSM510META, CarlZeiss) with FDA (green) and PI (red) filters. The percentage of pollen viability that was counted was an average of ten transgenic (n = 10) with three independent experiments. An *in-vitro* pollen germination test was performed using artificial liquid medium (10% Sucrose, 0.1 mg/ml Boric acid, 0.3 mg/ml calcium nitrate, 0.2 mg/ml magnesium sulfate, and 0.1 mg/ml potassium nitrate). Pollen germination images were taken on Leica microscope. Pollen germination (%) was an average of 10 lines (n = 10) with three replicates of the experiment.

### Transmission electron microscopy (TEM) and Scanning electron microscopy (SEM)

Anther samples of developmental stages from −5 to +6 stage as described by^46^ were washed with 1xPBS (pH 7.2) before fixing in 2.5% glutaraldehyde prepared in .1 M sodium cacodylate (Ladd Research) buffer (pH 7.2) for 2 h at 4 ^°^C. Samples were washed thrice, with 0.1 M sodium cacodylate buffer and post-fixed in 1% osmium tetraoxide for 2 h. Further, samples were washed with sodium cacodylate, dehydrated in acetone series (15–100%) and embedded in araldite-DDSA mixture (Ladd Research Industries, USA). After baking at 60 ^°^C, blocks were cut (60–80 nm thick) by an ultra-microtome (Leica EM UC7) and sections were stained by uranyl acetate and lead citrate. Analysis of sections was done under FEI Tecnai G2spirit twin transmission electron microscope equipped with Gatan digital CCD camera (Netherland) at 60 or 80 kV.

For SEM analysis stage 7 anthers of control (NTPH), 1371 transgenic, and F1 and F’1 were washed twice in 0.1 M Sodium cacodylate buffer and fixed in 2.5% glutaraldehyde and 4% paraformaldehyde overnight 4 ^°^C. Samples were washed thrice in 0.1 M Sodium cacodylate, 20 min each, and transferred to Osmium tetraoxide overnight; further, two washings in 0.1 M sodium cacodylate buffer were done, dehydration was conducted in acetone series, 15%, 30%, 60%, and 90% and 3 changes were made in 100% for 20 min each. Further samples were dehydrated till they reach critical point (CPD), anthers were ruptured, and pollens were taken on the stub adhesive, coated with gold particles (2 coating), and observed under the scanning electron microscope (FEG450 Quanta, Netherland).

### RNA extraction and quantitative real-time RT-PCR

Total RNA was isolated using Plant Spectrum Total RNA isolation kit according to manual instructions (Sigma –Aldrich, http://www.sigmaaldrich.com). After DNaseI treatment (Ambion Inc, Austin TX USA), RNA integrity was checked by electrophoresis and quantified by using using a NanoDrop^®^ ND-1000 UV-Vis spectrophotometer. 2 μg RNA was reverse transcribed using oligo(dT) primers and Superscript II RT (Invitrogen, Rockville, MD, USA) into first-strand c-DNA in a 20-μL reaction as per manual instructions. Quantitative real-time PCR was performed by Express SYBR^®^Green ER™ qPCR SuperMix Universal (Invitrogen) using the ABI 7500 Fast Real-Time PCR Detection System (Applied Biosystems). The gene-specific primers listed in Table S1 were utilized. UBQ10 was taken as an internal control. Relative expression was calculated from threshold cycle values^52^). Three independent qRT-PCR reactions were performed on different cDNA samples.

### Microtome and light microscopy

Tobacco anthers of developmental stage from −1 to +6^46^ were immediately fixed in Poly/LEM fixative (Polysciences, Inc. Cat# 16864) overnight at 4 ^°^C. Fixed anthers were dehydrated in ethanol series 50%, 70%, 85%, 95% and 100% for 1h each. Infiltration was done in 2:1, 1:1 and 1:0 of (ethanol: infiltration solution) for 1h each and embedded in resin (JB-4 Embedding Kit, Polysciences Inc. Eppelheim, Germany). A 4-μM-thick section were cut by using Leica microtome, stained with 0.1% toluidine blue O’ (Sigma Aldrich) and pictures were captured using a Nikon microscope. Pictures were adjusted using adobe paint (NET) software.

### Crossing strategies and F1 screening

The best expressing lines of transgenic 1370 were taken as the female parent and were crossed by the two best performing lines of 1372 and 1373 which were taken as male parents; F1 and F’1 were generated. Similarly 1371 transgenics were taken as the female parent and were crossed with the best performing 1372 and 1373 pollens; F_1_ and F’_1_ were generated. The F_1_/F’_1_ seeds were germinated on medium containing kanamycin (300 mg/litre) and hygromycin (50 mg/litre) on a petri-dish for 3 weeks. Plantlets of selected crosses were transferred into pots in a transgenic house.

## Additional Information

**How to cite this article**: Singh, S. P. *et al*. A novel male sterility-fertility restoration system in plants for hybrid seed production. *Sci. Rep*. **5**, 11274; doi: 10.1038/srep11274 (2015).

## Supplementary Material

Supplementary Information
