# Supplementary material for: A novel male sterility-fertility restoration system in plants for hybrid seed production
Source: Sci Rep. 2015 Jun 15;5:11274. doi: 10.1038/srep11274 (PMC4466886; doi:10.1038/srep11274)
Supplement: Supplementary Information [file srep11274-s1.pdf]

A novel male sterility-fertility restoration system in plants for hybrid seed production

Surendra Pratap Singh[1], Sudhir P. Singh[3], Tripti Pandey[1], Ram Rakshpal Singh[4], Samir V. Sawant[1, 2*]

Supplementary Information

Supplementary Figure S1. Schematic representation of expression cassettes and analysis of pollen fertility**.**
Supplementary Figure S2. Fluor-metric GUS expression analysis.
Supplementary Figure S3. Analysis of 1372 (A9:*COP1*) transgenic lines.
Supplementary Figure S4. Screening of best expressing transgenic lines.
Supplementary Figure S5. Screening of F1 lines.
Supplementary Figure S6. Pollen wall architecture in control (NTPH) and Beclin1 transgenic (1371).
Supplementary Figure S7. Pollen surface morphology in control (NTPH), Beclin1 transgenic (1371), F1 and F'1.
Supplementary Table S1. List of primers used in this study

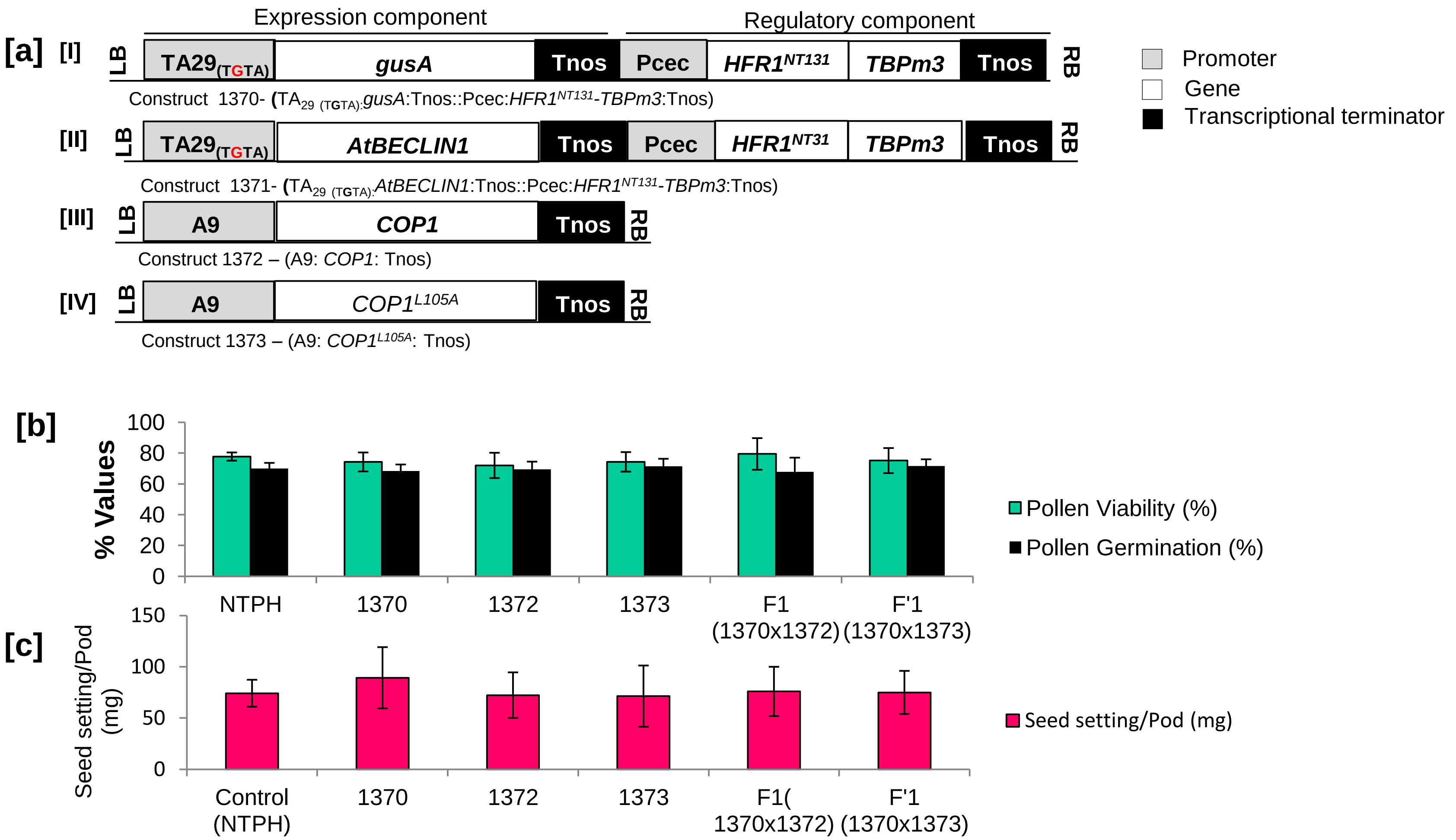

Fig. S1 **Schematic representation of expression cassettes and analysis of pollen fertility.**

[a] Construct 1370 with two transcriptional units; expression component ($TA_{29(TGTA)}$;*gusA*:Tnos) which is regulated by regulatory component (Pcec:*HFR1*^{NT131}-*TBPm3*:Tnos), cloned in LB and RB of binary vector pBI101**[I]**. Construct 1371 where *gusA* gene was replaced by Arabidopsis *BECLIN1/ATG6* in 1370 making two-component ES ($TA_{29\ (TGTA)}$;*AtBECLIN1*: Tnos:: Pcec:*HFR1*^{NT131}-*TBPm3*:Tnos) cloned in LB and RB of binary vector pBI101 **[II]**. Construct 1372 expressing COP1 using A9 promoter, cloned in LB and RB of modified pCAMBIA1300 binary vector **[III]**. Construct 1373 expressing COP1^{L105A} using A9 promoter, cloned in LB and RB of modified pCAMBIA1300 binary vector **[IV]**. [COP1^{L105A}= COP1 with mutation in the CLS to increase nuclear abundance, modified pCAMBIA1300= modified by replacing CaMV35S promoter by pNOS promoter to avoid leaky expression of COP1 due to bidirectional expression possibility by CaMV35S promoter]

Pollen viability, in-vitro pollen germination **[b]**, and seed setting per pod (in mg) **[c]** in control, 1370 , 1372, 1373 transgenic lines , F1 (1370x1372) and F’1 (1370x1373). Error bar represents SD of 10 independent replicates.

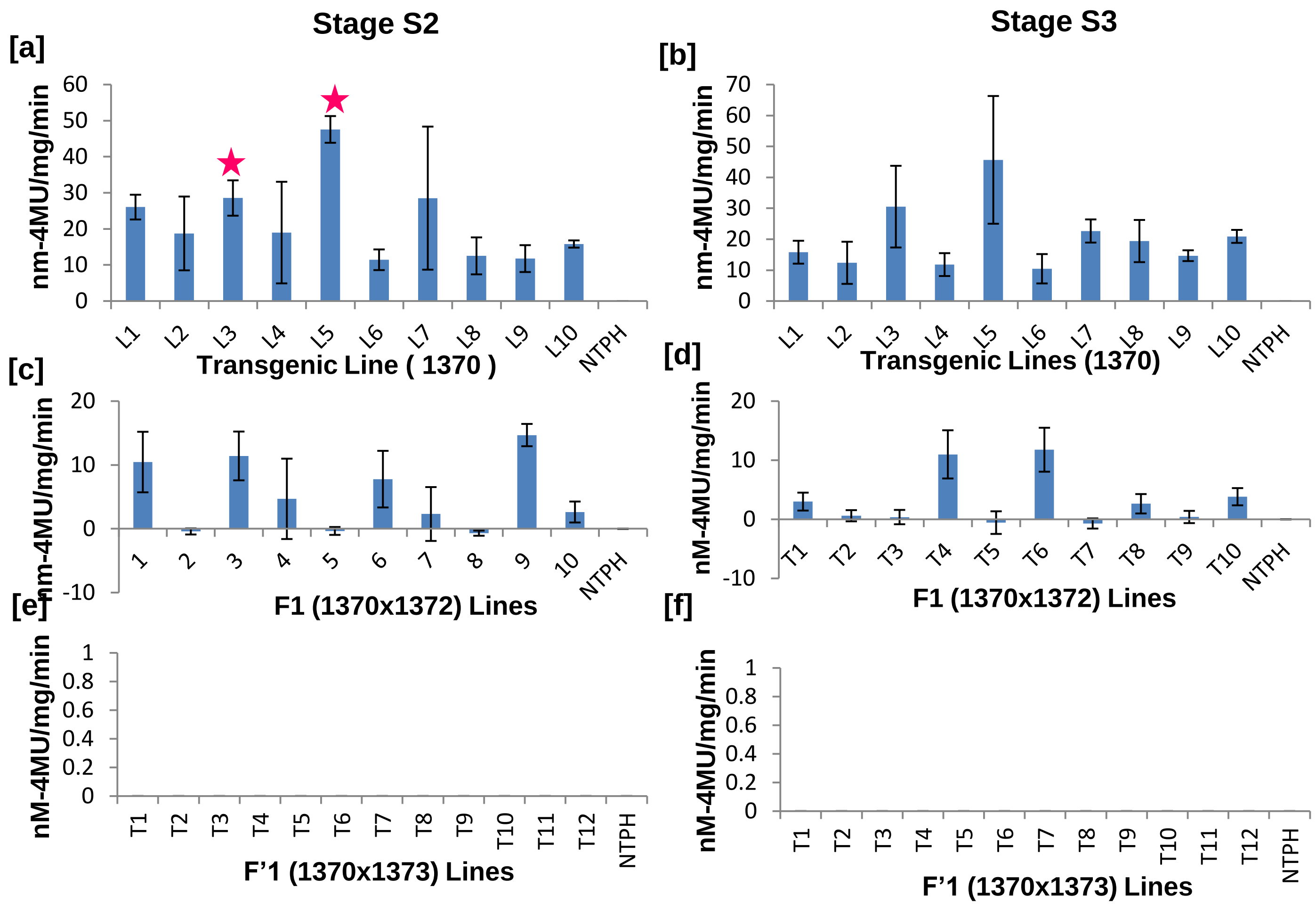

Supplementary Figure S2. Fluor-metric GUS expression analysis.

[a-b] GUS expression analysis in 1370 (**TA$_{29(m)}$**:*GUS*:**Pcec:***HFR1^{NT131}-TBPm3)* transgenic lines of anther developmental stage 2 [a] and 3 [b]. **[c-d]** GUS expression analysis in the F1 anther stage 2 [c] and 3 [d]. The F1 generated by crossing with 1370 (♀)line 3 (L3) and line 5 (L5) with 1372 (**A9:*COP1***) lines (♂) T13 and T33. **[e-f]** GUS expression analysis in the F'1 anther stage 2 [e] and 3 [f]. The F'1 generated by crossing with 1370 (♀)line 3 (L3) and line 5 (L5) with 1373 (**A$_9$:*COP1^{L105A}***) lines (♂) T1 and T3.
(The expression values are normalized with control (NTPH) values on their respective stages2 and 3. star indicates the lines of 1370 selected for crossing as female parent. Error bar represent three replicate , each with n=3)

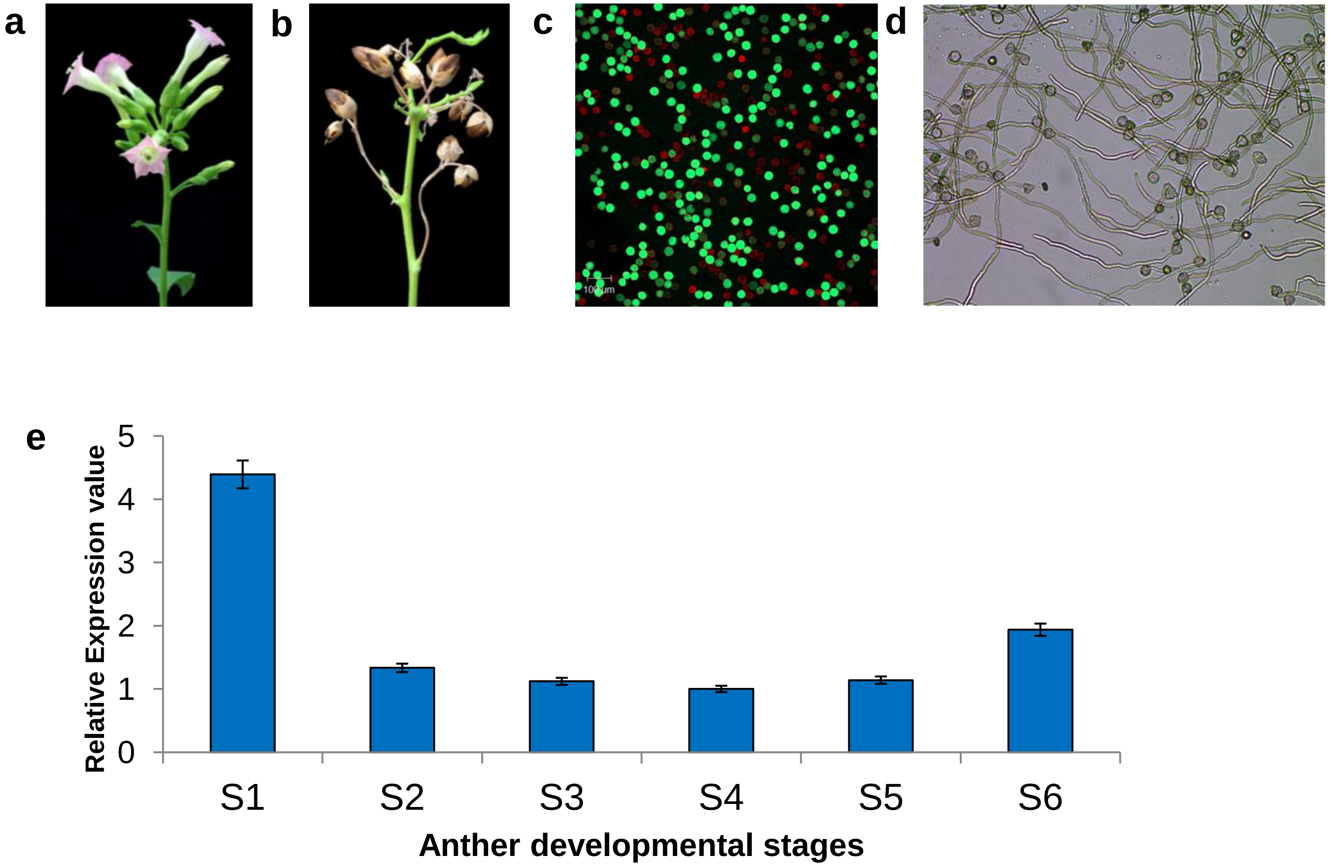

Supplementary Figure S3. Analysis of 1372 (A9:*COP1*) transgenic lines.
[a-d] Transgenic line of 1372 with normal flowering [a] and seed setting [b]. Pollens fertility as evaluated by pollen viability [c] and in-vitro pollen germination [d].[e] qRT-PCR analysis for relative expression of COP1 in the anther developmental stage 1 of1372 transgenic lines. COP1 expression was found to be specific to the stage 1 of the anther. Error bar represent SE of three independent Experiments. UBIQ10 used as internal control.

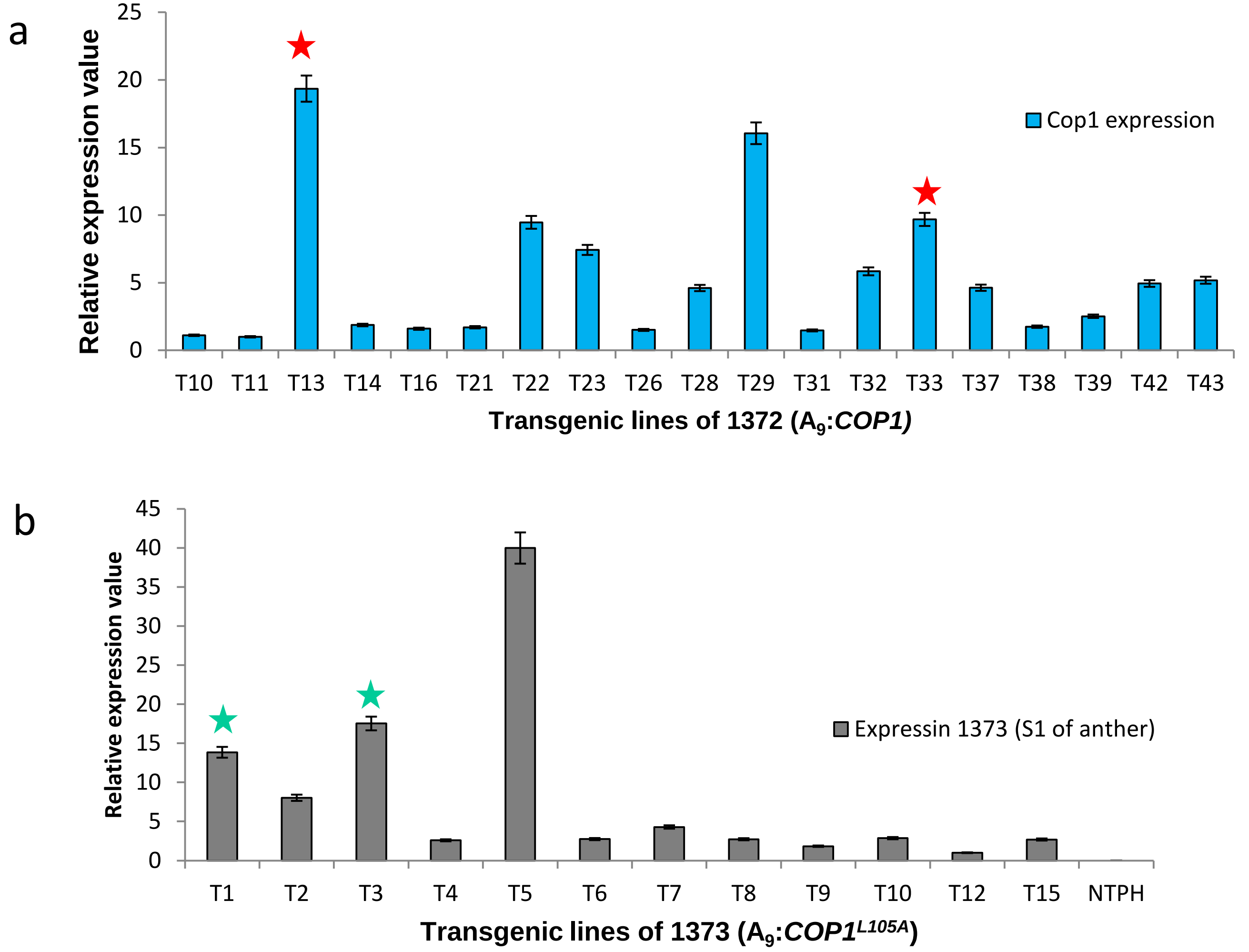

Supplementary Figure S4. Screening of best expressing transgenic lines.
[a] qRT-PCR analysis of stage 1 anther of different transgenic lines of 1372 (A_9:***COP1***), transgenic line 13 (T13) and 33 (T33) were selected for crossing (as male parent). **[b]** qRT-PCR analysis of stage 1 anther of different transgenic lines of 1373 (**A_9:*COP1*^{L105A}**), transgenic line 1(T1) and 3 (T3) were selected for crossing (as male parent). Error bar represents SE of three independent experiment (n=3) Tobacco *UBIQ10* taken as internal control.

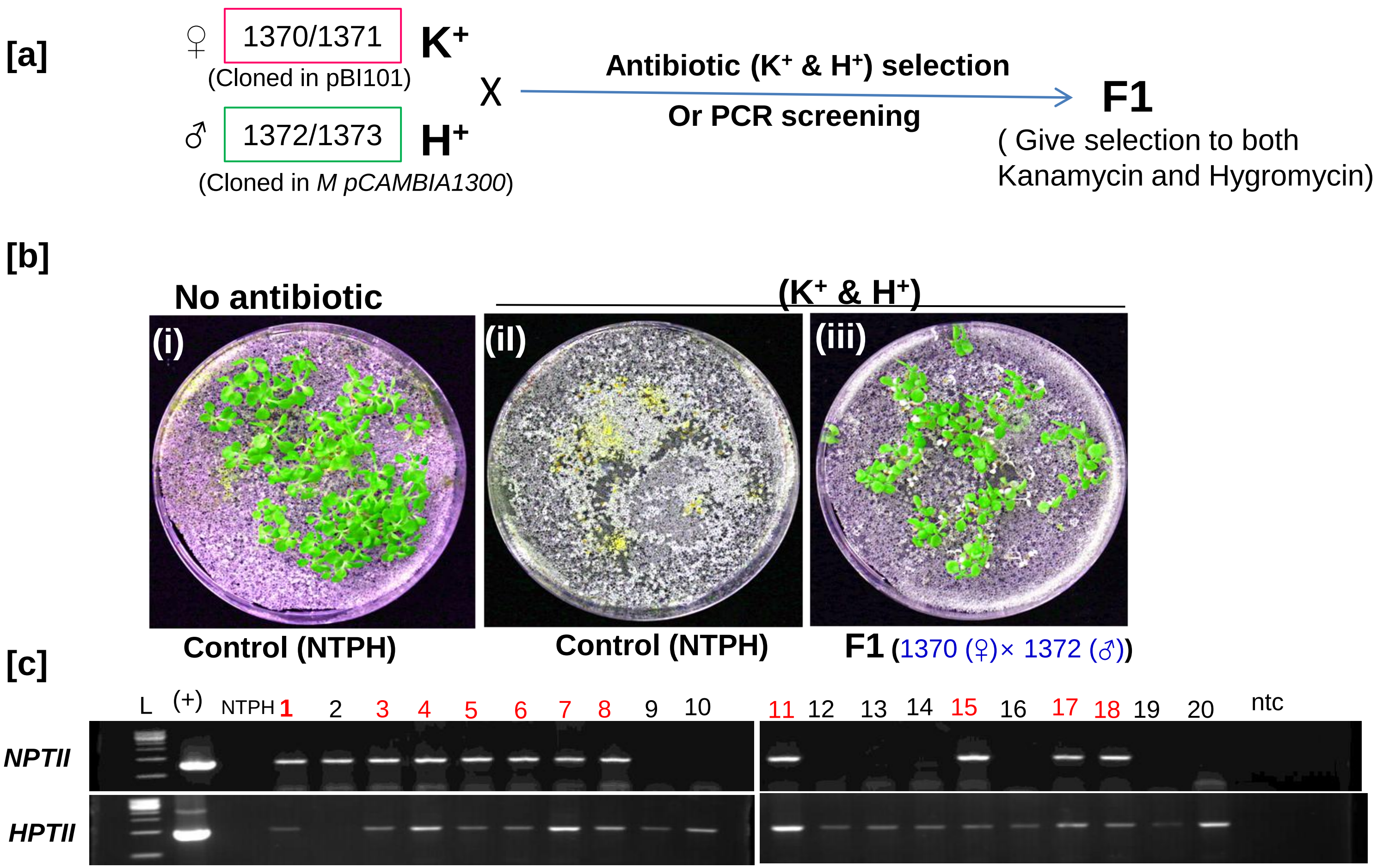

Supplementary Figure S5. Screening of F1 lines.

[a] Strategy or the F1 screening. **[b]** Screening of F1seeds on the double antibiotic selection ; Control (NTPH) seeds were sown without antibiotic selection (i) and with double selection of K^+ and H^+ (ii), while F1 seeds germinated on the double selection (iii).**[c]** PCR screening of F1s; F1 seeds obtained were sown directly in the soil and genomic DNA was isolated from their seedlings, slandered PCR screening was performed using *nptII* and *hptII* specific primer . F1 lines giving nptII and HptII positive amplification are the F1s. Positive control taken were vector construct and ntph as negative control while non-templet control (ntc) as null control.

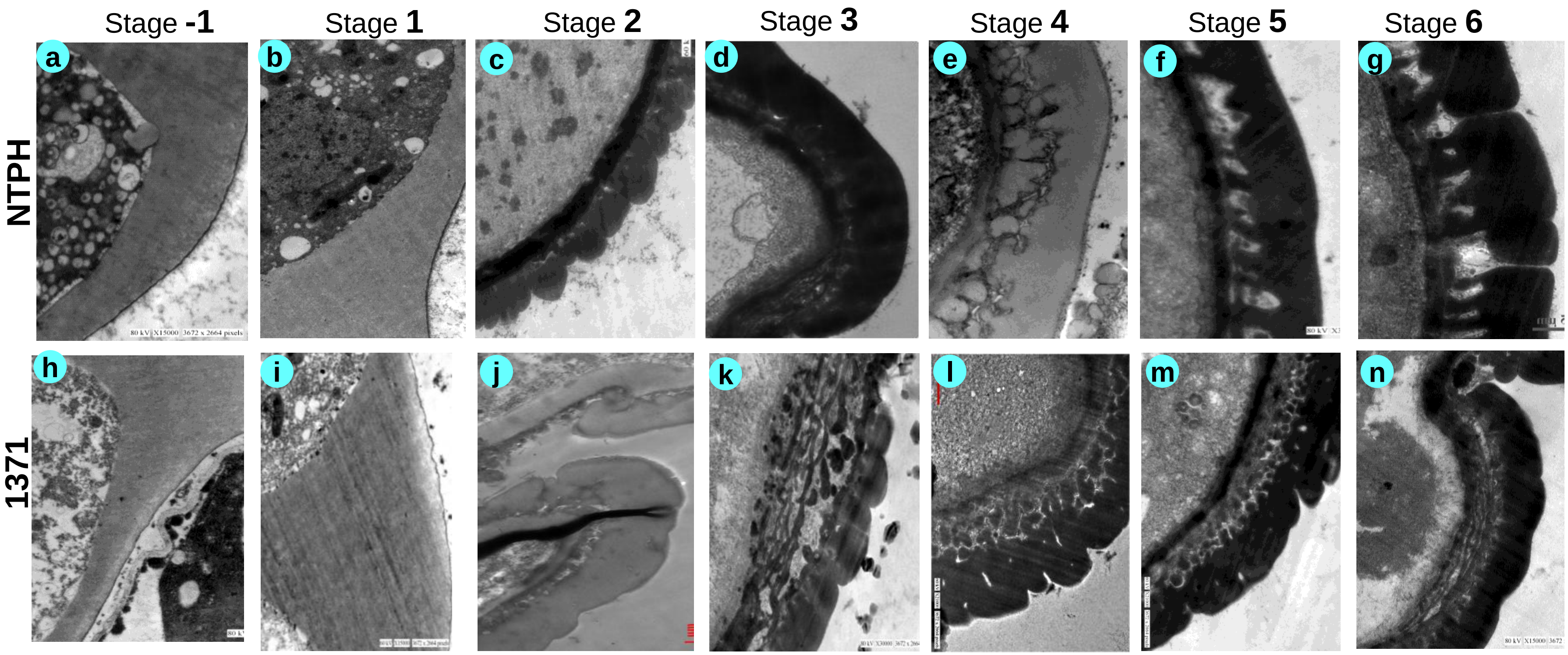

Supplementary Figure S6. Pollen wall architecture in control (NTPH) and Beclin1 transgenic (1371).
Stage -1 pollens covered with callose deposition in both control [a] and 1371 transgenic [b] which is consistent in Stage 1 the ([b] and [i]). Stage 2 pollen showing exine and intine development in control [c] while undecomposed callose was remain deposited over the 1371 [j]. Exine sculpturing was progressively developed in control in stage 3 [d], stage 4 [e], stage 5 [f] and stage 6 [g] resulting in the well developed wall architecture, whereas in 1371 transgenic defective pollen wall development started in stage 3 [k], followed by stage 4 [l], 5 [m] and complete collapse in stage 6 [n].

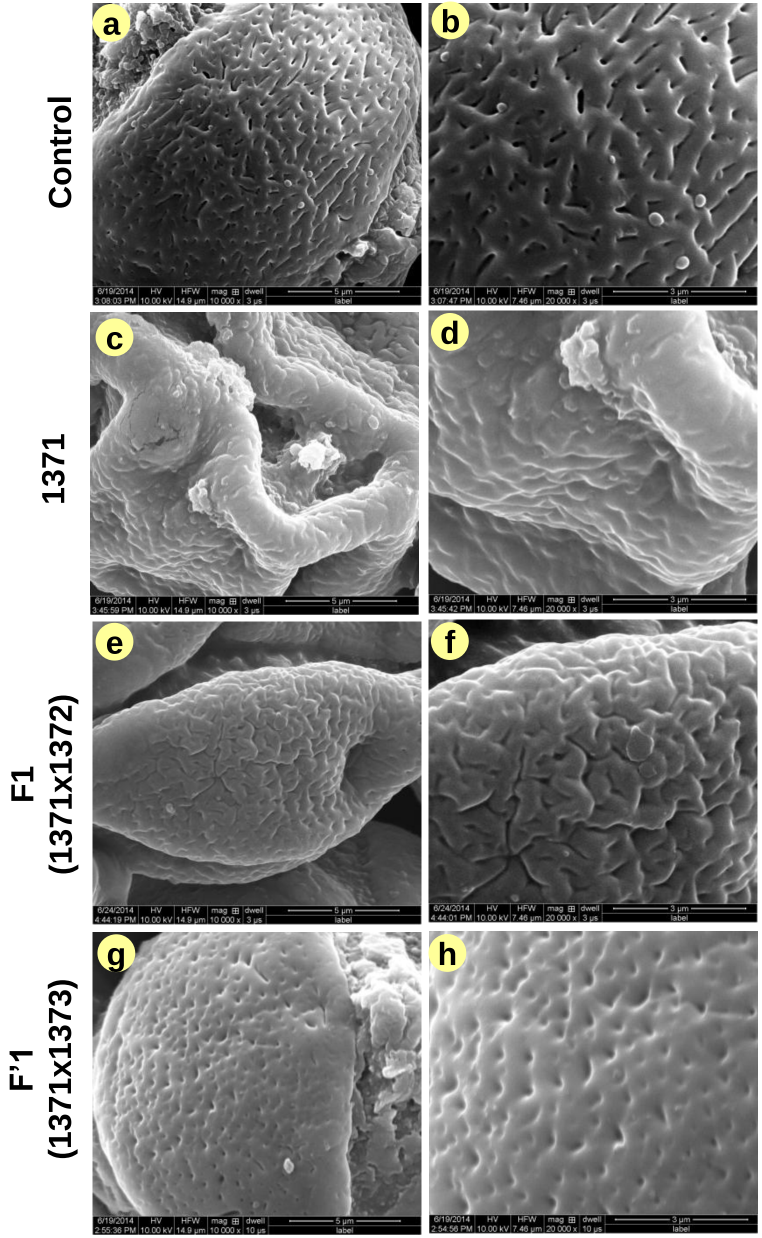

Supplementary Figure S7. Pollen surface morphology in control (NTPH), Beclin1 transgenic (1371), F1 and F'1. Pollen surface of control showing reticulated exine architecture **([a]** and **[b]).** In 1371 transgenic deformed, shrunken pollen wall without pore was found **([c]** and **[d]**) whereas some degree of pollen deformation was limited in F1 (**[e]** and **[f]**). F'1 showing surface architecture (**[g]** and **[h]**)similar to control. Bar= 5μm in [a], [c], [e], and [g] and 3μm in [b], [d], [f], and [h].

Supplementary Tables

Table_S1 : List of primers used in this study				
**S.No.**	**Gene/target**	**Primers**	**restriction site**	**Used for**
1	TA29	Forward: 5’-CGCGGATCCAGATCTTCCAACACCATTTACTCCAAGGG-3’	*BamH*I	promoter amplification
		Reverse: 5’-CTAGTCTAGATTTAGCTAATTT CTTTAAGTAAAAACT-3’	*Xba*I	
		Forward: 5’ TGTATGTCTTGTGCTCTGTATATGCCCTTGTGGTG 3’		Site directed mutagenesis
		Reverse: 5’ CACCACAAGGGCA TATACAGAGCACAAGACATACA 3’		
2	*Beclin1/ATG6*	Forward: 5’-CTAGTCTAGAATGAGGAAAGAGGAGATTCCAGA-3’	*XbaI*	Gene amplification
		Reverse: 5’-CGTCGAGCTCCTAAGTTT TTTTACATGAAGGCTTA-3’	*SacI*	
		Farward: 5’AGGGCATTCCTCCACGTC3’		qRT-PCR
		Reverse: 5’ AAGAGACA GATTGTGAGAACCACCA 3’		
3	*HFR1*	Forward: 5’CCATCGATATGTCGAATAATCAAGCTTTCATGG 3’	*ClaI*	Gene amplification
		Reverse: 5’CCATCGATTCTTGTAAACTCCTCCGATTCATC3’	*ClaI*	
4	COP1	Forward: 5’CCGCTCGAGATGGAAGAGATTTCGACGGATCC3’	*XhoI*	Gene amplification
		Reverse: 5’CGAGCTCTCACGCAGCGAGTACCAGAACTT 3’	*SacI*	
		Forward: 5'GCTTTACCCTAATTTCGCGGCCCGATAAGCTATTGAAGAAAACTTC 3'		Site directed mutagenesis
		Reverse: 5'GTTTTCTTCAATAGCTTATCGGCCGCGAAATTAGGGTAAAGCTG 3'		
		Forward: 5' TAATTTCTTGCTCGATAAGGCAGCGAAGAAAACTTCAGCTCGGC 3'		
		Reverse: 5' CGAGCTGAAGTTTTCTTCGCTGCCTTATCGAGCAAGAAATTAGG 3'		
		Forward: 5’AAGCGGCGGTTCTGAGATT3’		qRT-PCR
		Reverse: 5’ACCACAAGCCGTGAGGAAAG3’		
		Forward: 5'-AAGCGGCGGTTCTGAGATT-3'		qRT-PCR (COP1m)
		Reverse: 5'-ACCACAAGCCGTGAGGAAAG-3'		
5	A9	Forward: 5’-ACGCGTCGACTCTAGACATAACGGTGAGAGTTAA-3’	*SalI*	promoter amplification
		Reverse: 5’-TCCCATCGATTCTAATTAGATACTATATTGTTTGTAC-3’	*ClaI*	
6	*UbiQ10*	Forward: 5’CCACGGAGACGGAGGACAA3’		qRT-PCR
		Reverse: 5’GAAGCA GCTCGAGGATGGAA3’		
